# The Genetic Landscape of Cutaneous Lupus Erythematosus

**DOI:** 10.3389/fmed.2022.916011

**Published:** 2022-06-02

**Authors:** Henry W. Chen, Grant Barber, Benjamin F. Chong

**Affiliations:** Department of Dermatology, University of Texas Southwestern Medical Center, Dallas, TX, United States

**Keywords:** cutaneous lupus erythematosus, DNA, RNA, genetic polymorphism, microarray, inflammation, apoptosis, fibrosis

## Abstract

Cutaneous lupus erythematosus (CLE) is an autoimmune connective tissue disease that can exist as a disease entity or within the context of systemic lupus erythematosus (SLE). Over the years, efforts to elucidate the genetic underpinnings of CLE and SLE have yielded a wealth of information. This review examines prior studies investigating the genetics of CLE at the DNA and RNA level and identifies future research areas. In this literature review, we examined the English language literature captured within the MEDLINE and Embase databases using pre-defined search terms. First, we surveyed studies investigating various DNA studies of CLE. We identified three predominant areas of focus in HLA profiling, complement deficiencies, and genetic polymorphisms. An increased frequency of HLA-B8 has been strongly linked to CLE. In addition, multiple genes responsible for mediating innate immune response, cell growth, apoptosis, and interferon response confer a higher risk of developing CLE, specifically TREX1 and SAMHD1. There was a strong association between C2 complement deficiency and CLE. Second, we reviewed literature studying aberrations in the transcriptomes of patients with CLE. We reviewed genetic aberrations initiated by environmental insults, and we examined the interplay of dysregulated inflammatory, apoptotic, and fibrotic pathways in the context of the pathomechanism of CLE. These current learnings will serve as the foundation for further advances in integrating personalized medicine into the care of patients with CLE.

## Introduction

Cutaneous lupus erythematosus (CLE) is a heterogeneous autoimmune disease that can be skin-limited or exist within the context of systemic lupus erythematosus (SLE). With the advancement of genetic sequencing technology at the DNA and RNA level, more dysregulated pathways and gene networks that contribute to the development of CLE have been identified. Specifically, differential expression of key genes involved in various pathways, such as inflammation, apoptosis, and immunity has revealed a complex, heterogeneous picture. These new gene expression profiles offer the opportunity to further delineate classification subsets of CLE and potentially predict prognosis, such as response to treatments and progression to systemic disease (1, 2).

Up until recently, the development of new therapies for CLE has been stymied by an incomplete understanding of the underlying pathophysiology of CLE. Given the importance of understanding the genetic landscape of CLE, we performed a literature review to summarize studies examining DNA and RNA genetic aberrations in CLE.

## Methods

This was a review of the English-language literature captured within the MEDLINE and Embase databases using pre-defined search terms (Supplementary Table 1) from inception through 7 February 2022. Two independent reviewers (H.W.C. and G.B.) reviewed all studies, and a third reviewer (B.F.C.) resolved any discrepancies. Inclusion criteria were original studies, case series, and case reports related to CLE in humans in the English language. Articles underwent title and abstract screening with a subsequent full-text review. Articles were included if their findings were pertinent to DNA or RNA in the context of CLE. Reviews, conference abstracts, editorials, and all non-peer-reviewed findings were excluded from this review. In total, 1,253 studies were identified for screening, and 105 studies were ultimately included for final review after applying inclusion and exclusion criteria (Supplementary Figure 1).

## Results

### DNA

Studies examining DNA have long been performed to better our understanding of cutaneous lupus. Three major themes emerged from our review of these studies, such as HLA profiling, complement deficiencies, and genetic polymorphisms.

#### Human Leukocyte Antigen Genes Have Been Associated With CLE and Its Subtypes

Human leukocyte antigen (HLA) is quintessential in the differentiation of self and non-self and plays a strong role in autoimmunity. HLA profiling studies have been performed in small groups of patients with CLE and controls to better understand genetic variations. Fowler et al. (3) found an increased frequency of HLA-DRw6 among both White and Black patients with CLE. In further studies, HLA-B8 has repeatedly been found to be increased among patients with discoid lupus erythematosus (DLE) and subacute cutaneous lupus erythematosus (SCLE) (4–7). Bielsa et al. (4) found an increased frequency of HLA-B8 and HLA-DR3 along with a decreased frequency of HLA-DR5 in patients with annular SCLE when compared with controls. Another study of 11 Finnish patients with SCLE and 23 controls showed that HLA-DR3, HLA-B8, and HLA-DR2 were higher in patients with SCLE vs. controls (7). Fischer et al. (8) found the HLA-DQA1*OI02 allele was significantly increased among 26 patients with chronic CLE (CCLE) vs. healthy controls. The HLA-DQA1 alleles have also been studied in neonatal lupus erythematosus (NLE), with mothers of seven NLE children all carrying at least one DQA1 allele with glutamine at position 34 of the first domain compared with just 44% of controls (9). Another study of 28 patients with DLE showed a higher frequency of HLA-DRB1*04 (10). These studies highlight the inherent importance of HLA variations in CLE, though many are limited by small sample sizes. Larger studies, such as genome-wide association studies, in diverse populations, would help elucidate these variations.

#### Deficiencies in the Complement Cascade Contribute to Cutaneous Lupus Erythematosus Pathogenesis

The complement cascade is indispensable in mediating phagocytosis and inflammation. C1q is a subcomponent of C1 comprised of three heterotrimeric subunits (C1qA, C1qB, and C1qC). In a cohort of 19 White patients with SCLE, homozygous C1qA A > G transition mutation in exon 2, which results in a synonymous mutation, was found to occur more frequently in patients with SCLE relative with healthy controls (11). Despite no alterations in the protein sequence, decreased C1q protein was still observed. Another case study identified a homozygous G > C transversion mutation in C1qC exon 1, resulting in a Gly61Arg mutation (12). Multiple case studies have identified C2 deficiency in patients with SCLE and DLE (13–15). The gene encoding C2 lies within the major histocompatibility complex and is thus linked with HLA-A10, -A25, -B18, -DR2, and -Dw2. Agnello et al. (16) examined the pedigrees of four patients with DLE and found a partial genetic deficiency of C4 in patients carrying the null C4 allele B*QO. Given the important function of the complement cascade in mediating phagocytosis and inflammation, further studies on the complement system’s role in the pathogenesis of CLE would be beneficial.

#### Genetic Polymorphisms Have Been Featured in Familial Chilblain Lupus and Other Cutaneous Lupus Erythematosus Subtypes

Genetic polymorphisms have also been a frequent focus of study for lupus. Table 1 summarizes prominent ones that have been identified, such as *TREX1*, *SAMHD1*, and tumor necrosis factor *(TNF)*. *TREX1* has been identified as a significant factor in familial chilblain lupus. Günther et al. (17) identified a potential mutation hotspot for *TREX1* where 4 of 6 families affected by familial chilblain lupus all present the same mutation. Günther et al. (18) also linked *TREX1* with the upregulation of type I interferon (IFN) activity in familial chilblain lupus. Another case report of a family with familial chilblain lupus revealed that three affected individuals all carried the same heterozygous mutation (19). Heterozygous mutations in *SAMHD1* have also been identified in patients with familial chilblain lupus independently of *TREX1* mutations, as reported by Ravenscroft et al. (20). Further, Linggonegoro et al. (21) found a deletion of the *SAMHD1* gene’s initiator region in a child with familial chilblain lupus, who did not show an increased IFN signature. *TNF* is another gene investigated extensively in lupus. Mutations involving this gene seem to be distinctly associated with SCLE rather than DLE (6, 22, 23). Millard et al. (6) reported a significantly higher frequency of *TNF-*α (−308 G/A) single nucleotide polymorphism (SNP) among 36 patients with SCLE compared with 49 patients with DLE and 102 healthy relatives of patients with lupus. Similarly, *TNF-*α (−308 G/A) SNP was found to be a significant risk factor among 192 patients with SLE, but not among 56 patients with DLE (23). Studies on *ITGAM* polymorphisms compared in patients with DLE and SLE have shown conflicting results. One study of 21 patients with DLE and 35 patients with SLE showed polymorphisms of *ITGAM* among patients with SLE but not among patients with DLE (24). However, another study of 177 patients with DLE and 85 patients with SLE found *ITGAM* polymorphisms to be 3-fold greater in DLE compared to controls and five times greater than in SLE (25).

**TABLE 1:** Genetic polymorphisms investigated in cutaneous lupus studies and their functions.

Gene	Function	Relevance to CLE and its subtypes
*C1QA*	Encodes the C1q subcomponent of the C1complement system	SNP of gene has significant association with SCLE compared to normal (11)
*CSNK2B*	Subunit of a protein kinase for regulation of metabolic pathways and DNA replication and transcription and mRNA translation	Has SNP strongly associated with CLE (26)
*CTLA4*	Protein involved in signaling T cell inhibition	Higher disease risk for DLE from haplotype variation (29)
*HLA-DRB3*	Cell surface molecule for antigen presenting cells. Presents extracellular protein derivatives for immune response	Independent SNP with a high association with CLE (26)
*HLA-DQA1*	Cell surface molecule for antigen presenting cells. Presents extracellular protein derivatives for immune response.	Has SNP with strong association with CLE (26)
*IL10*	Cytokine produced by monocytes. Affects immunoregulation and inflammation and regulated JAK-STAT pathway.	SNP associated with DLE but not SLE (23)
*IRF5*	Transcription factor with roles in virus-mediated activation and regulation of cell growth, differentiation, apoptosis and immune activity	SNP associated with increased risk for DLE and SCLE (29)
*ITGAM*	Integrin important for adhering neutrophils and monocytes to endothelium	Polymorphisms in DLE three-fold greater than normal and five-fold greater than SLE (25). Significantly greater allele frequency in SLE compared to normal but no allele variation in DLE (24).
*IZKF*	Protein involved with remodeling chromatin. Potential susceptibility gene for SLE	Two SNPs with nearly significant association to CLE are approximately ∼36kB and 41kB upstream of the gene (26)
*MICA*	Stress induced cell surface protein recognized by delta T cells in the intestinal epithelium	Believed to be associated with SNP ∼27kB away that is strongly associated with CLE (26)
*MICB*	Stress induced cell surface protein which activates NK cells and CD8 T cells	SNP for this gene is strongly linked to another SNP associated with CLE (26)
*MSH5*	Protein involved in mismatch repair associated with crossing over during meiosis. Also associated with radiation-induced apoptosis.	Has SNP strongly associated with CLE (26)
*RPP21*	Protein subunit of ribonuclease P. Processes 5’ head for tRNA	Cluster of 3 SNPs with strong association to CLE (26)
*SAMHD1*	Protein involved in innate immunity and response to infection. Plays a role in TNF-α signaling.	Mutation of the gene linked with familial chilblain lupus (20, 21)
*STAT4*	Transcription factor essential for mediating IL-12 response and helper T cell differentiation	SNP of this gene has an association with both DLE and SLE compared to normal (24)
*STING*	Transmembrane protein that is a major regulator of innate immune response to viral and bacterial infections	Heterozygous gene mutation found in five family members with familial chilblain lupus (30)
*TLR7*	Toll-like receptor protein for pathogen recognition and activation of innate immunity	Two SNPs with frequencies in SLE patients two times greater than normal. No significant difference in DLE (27)
*TNF*	Cytokine secreted by macrophages that regulates cell proliferation, differentiation and apoptosis	Greater allele variation in SCLE and SLE patients than DLE and normal patients (6, 22, 23)
*TNXB*	Glycoprotein associated with the extracellular matrix that functions in matrix maturation during wound healing	Significantly greater allele frequency in SLE compared to normal but no allele variation in DLE (24)
*TRAF3IP2*	Protein involved with regulation cytokine response and plays a central role in innate immune response to pathogens, inflammation, and stress	Novel SNP found in four siblings with DLE (28)
*TREX1*	Protein associated with DNA polymerase proofreading. Has exonuclease activity that plays a role in DNA repair	Mutation of the gene linked with familial chilblain lupus (17–19)
*TRIM39*	Protein of the tripartite motif family. Believed to have a role in apoptosis but not fully studied	Cluster of three SNPs with strong association to CLE (26)
*TYK2*	Protein is part of JAK family. Is a component of type I and type III interferon signaling pathways	SNP with increased risk of DLE but not SCLE (29)

To date, only Kunz et al. (26) have performed a genome-wide association study specifically examining patients with CLE. A comparison of 183 German patients with CLE, including DLE, SCLE, and lupus erythematosus tumidus (LET) subtypes, against healthy controls with a validation set of Finnish patients with DLE (*n* = 177) and SCLE (*n* = 42), revealed 62 SNPs predominantly on chromosome 6 in the major histocompatibility complex region. The presence of SNPs associated with apoptosis and inflammation (i.e., *TRIM39/RPP21*) and previously described in SLE (i.e., *HLA-DQA1, MICA/B*, and *IZKF*) suggests unique and overlapping genetic underpinnings of CLE and SLE.

In summary, numerous genes were found to have SNPs that were associated with a greater risk of CLE and SLE, such as *TLR7, TRAF3IP2*, *TYK2*, *IRF5*, *IL10*, *C1QA*, and *STAT4* (11, 23, 24, 27–29). Table 1 summarizes other additional genes whose polymorphisms are distinctly different in CLE and SLE groups (23, 24, 28–30).

### RNA

**FIGURE 1:**
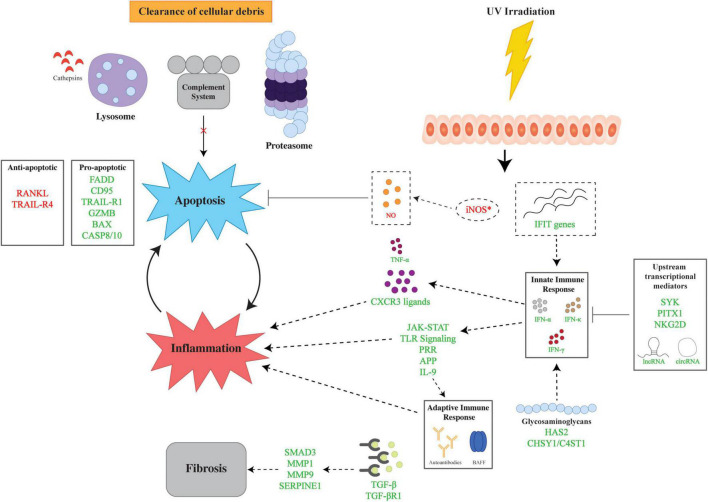
Overview of major pathways in the genetic pathophysiology of cutaneous lupus erythematosus (CLE). The genetic pathophysiology of CLE encompasses three main pathways: inflammation, apoptosis, and fibrosis. UV irradiation is a major environmental insult that serves to initiate CLE pathogenesis by aberrant expression of iNOS and *IFIT* genes which lead to apoptosis and inflammation, respectively. The inflammatory response is mediated by the innate and adaptive immune response *via* IFNs and autoantibodies. Upstream transcriptional mediators regulate the innate immune response *via* transcriptional factors and non-coding RNAs. Glycosaminoglycans also modulate the inflammatory response *via* the upregulation of HAS2 and CHSY1/C4ST1. Apoptosis is facilitated by dysregulation of pro- and anti-apoptotic genes in tandem with impaired cellular clearance *via* aberrations in the lysosome, proteasome, and complement. Finally, fibrosis is mediated by TGF-β and downstream upregulation of effector molecules. Lines with bars indicate inhibition. Arrows depict subsequent effect of molecule or process. Green text signifies upregulation and red text signifies downregulation. APP, antigen processing and presentation; BAX, Bcl-2 Associated X-protein; GZMB, Granzyme B; C4ST1, carbohydrate sulfotransferase 11; CASP8/10, caspase 8/10; CD95, Fas; CHSY1, chondroitin sulfate synthase 1; circRNAs, circular RNAs; CXCR3, C-X-C motif chemokine receptor 3; FADD, Fas Associated via Death Domain; HAS2, hyaluronan synthase 2; IFIT, interferon induced proteins with tetratricopeptide repeats; IFN-α, interferon alpha, IFN-γ, interferon gamma; IFN-κ, interferon kappa; JAK-STAT, Janus kinase signal transducer and activator of transcription; lncRNAs, long non-coding RNAs; MMP1, matrix metallopeptidase 1; MMP9, matrix metallopeptidase 9, NKG2D, natural killer group 2D; PITX1, paired like homeodomain 1; RANKL, receptor activator of nuclear factor kappa-beta ligand, SERPINE1, Serpin Family E Member 1; SMAD3, SMAD Family Member 3, SYK, spleen tyrosine kinase; TGF-β, transforming growth factor beta; TGF-βR1, transforming growth factor beta receptor 1; TNF-α, tumor necrosis factor alpha; TLR, toll-like receptor; TRAIL-R1, TRAIL Receptor 1; TRAIL-R4, TRAIL Receptor 4; UV, ultraviolet.

Understanding the genetic aberrations at the DNA level serves as a foundation for examining the changes in the CLE transcriptome. The interplay of multiple pathways, namely, inflammation, apoptosis, and fibrosis, lays the framework for the pathomechanisms behind CLE (Figure 1). Herein, we describe the major contributors to CLE pathogenesis identified in gene expression analyses.

#### UV Irradiation Is a Major Initiator in the Pathogenesis of Cutaneous Lupus Erythematosus

UV irradiation has been thought to play a key role in the development of CLE, specifically due to the induction of autoantigens. After UV irradiation, nitric oxide is synthesized by nitric oxide synthases, such as inducible nitric oxide synthase (iNOS), and functions to protect cells, such as keratinocytes, from apoptosis (31, 32). Early work showed abnormal iNOS gene expression in the skin of patients with CLE patients who demonstrated delayed kinetics of iNOS induction by 72 h relative to controls (33). UVB irradiation induces chemokines, such as CXCR3 ligands CXCL9, CXCL10, and CXCL11, necessary to orchestrate the innate and adaptive response central to the immunopathogenesis of CLE (34). More recently, Katayama et al. (35) showed upregulation of the *IFIT* gene family, *HLA-DPA1*, and normal UV response genes (i.e., nucleic acid binding and erythematous reactions) in CLE skin relative to healthy skin. The *IFIT* gene family has subsequently been shown to be the top hub genes in bioinformatics analysis of DLE skin (36). This inflammatory response is mediated by IFNs with greater concordant elevations in IFN-α levels in SCLE relative to DLE.

#### Innate and Adaptive Immune Responses Drive Inflammation in Cutaneous Lupus Erythematosus Pathogenesis

For many years, unfettered inflammation secondary to dysregulated Th1 axis has been understood to be at the heart of CLE pathogenesis. Early studies using reverse-transcriptase PCR identified the potential role of type 1 cytokines and inflammation pathways. Patients with DLE without SLE were found to have increased expression of IFN-γ and IL-2 (37). An examination of the T-cell cytokine profile in CLE showed an upregulation of IFN-γ but also IL-5, indicating a possible role for Th2 cells (38). In the context of the B7-CD28 pathway, the importance of T-cells in the pathogenesis of CLE is underscored by findings of B7-1 and B7-2 RNA expression primarily in the dermis of patients with DLE, SCLE, and SLE (39). Microarray experiments comparing DLE to psoriasis confirmed a predominant Th1 signature and no Th17 signature, which is a hallmark of psoriasis (40). Bioinformatics analysis of gene networks notes an overlap of Th1 skewing of DLE with sarcoidosis (41). When CLE subtypes are compared, DLE and SCLE gene expression predominantly had a type I IFN signature, but DLE had a relatively increased expression of Th1-related cytokines (42).

Innate immune response functions upregulated by the Th1 phenotype include JAK/STAT signaling, toll-like receptor signaling, pattern recognition receptors, and antigen processing and presentation. Microarray and RNA-sequencing experiments consistently demonstrate upregulation of these inflammatory pathways in CLE skin (43–46). Recently, JAK/STAT upregulation in CLE has been the focus of targeted therapies, with JAK1-specific inhibition being explored as a promising approach for the treatment of CLE (47, 48). Enhanced toll-like receptor-dependent and pattern recognition receptor pathways contribute to both innate and adaptive immune responses in CLE (45). While often overlooked, only one study has examined the glycome of CLE given the function of glycosaminoglycans in mediating inflammation by acting as pathogen-associated molecular patterns (49, 50). Upregulation of hyaluronan and chondroitin sulfate *via HAS2* and *CHSY1*/*C4ST1*, respectively, provides some evidence by which glycosaminoglycans participate in the characteristic inflammatory response of CLE. Finally, in a study by Zhu et al. (1), a unique machine learning approach leveraging modular analysis uncovered large heterogeneity in CLE, but central themes of Th1 dysregulation and interferon activation were largely preserved in identified clusters.

The adaptive immune response is also important in the pathogenesis of CLE *via* dysregulation in antigen presentation, activation of B-cells, and autoantibody production (36). Moreover, key players, such as IL-9 and B-cell activating factor (BAFF) may be important in CLE progression to SLE and the distinction of CLE from SLE. Elevated *IL9* expression has been linked to production of autoantibodies in lupus-prone mice (51) and in skin of CLE patients who progressed to SLE versus those who did not (52). Similarly, our group found that higher BAFF mRNA and protein levels in patients with DLE with SLE than in those without SLE (53). Taken together, these findings suggest intricate interactions between the innate and adaptive immune response in mediating CLE pathogenesis and facilitating progression to SLE.

#### Upstream Regulation Impacts the Degree of Inflammatory Signatures in Cutaneous Lupus Erythematosus

Upstream mediators of inflammation have also been investigated. Spleen tyrosine kinase (*SYK*) is known to be a mediator of multiple innate and adaptive immune responses (54). Gene expression analysis *via* microarray of DLE (*n* = 7) and SCLE (*n* = 5) skin revealed upregulation of *SYK* and multiple *SYK*-regulated innate immune-related genes relative to healthy skin (*n* = 5) (55). Vorwerk et al. (56) postulate NKG2D, an immune receptor on NK cells and a subset of CD8 + T cells, contributes to CLE, giving upregulation on whole transcriptome RNA sequencing. However, NKG2D plays a selective role in autoimmune disease, and further mechanistic studies are required to understand its role in CLE (57). Recent RNA-sequencing data of SLE skin have identified another transcription factor, *PITX1*, which facilitates hypersensitive responses to type I IFNs in lupus keratinocytes (58). Finally, RNA-sequencing analysis of long non-coding RNAs (lncRNAs) and circular RNAs (circRNAs) was differentially expressed in patients with DLE and correlated with inflammatory immune response-related genes on coding and non-coding gene network analysis (59). Further functional studies of lncRNAs and circRNAs are required to fully understand their biological function.

#### Upregulated Cytokines and Chemokines Enhance Inflammatory Response in Cutaneous Lupus Erythematosus

One member of the type I IFN family, IFN-α, has had a well-established signature within SLE patients with skin involvement (60). In CLE, IFN-α is upregulated in both lesional and non-lesional skin (61). IFN-α recently has been shown to promote the adherence of *Staphylococcus aureus* with CLE and SLE keratinocytes (62). Thus, in concert with dysregulation of barrier proteins, such as filaggrin, IFN-α has been mechanistically implicated in the colonization of CLE lesions. In addition to IFN-α, IFN-κ has also been important in the dysregulation of CLE keratinocytes (58, 63, 64). Responsiveness to hydroxychloroquine therapy has been associated with an increased type I IFN signature, while high TNF-α was associated with response to adjunct quinacrine (2).

Upregulated expression of chemokines CXCL9, CXCL10, and CXCL11 and its receptor CXCR3 is a hallmark of CLE (65). These chemokines exert their effect on CXCR3-expressing cells and orchestrate the Th1 immune response by promoting Th1 cell migration (34, 48, 66). These chemokines have been repeatedly shown to facilitate interface dermatitis (66). CXCL9 and CXCL10 expression were strongly correlated with IFN-γ expression in DLE (*n* = 15), SCLE (*n* = 11), and LET (*n* = 21) skin. (67). Novel bioinformatics approaches have shown that these chemokines as key genes are involved in CLE (68).

#### Apoptosis Perpetuates Inflammation

Apoptosis is broadly comprised of the extrinsic and intrinsic pathways. Current evidence suggests increased activity of the extrinsic apoptotic pathway *via* the upregulation of the TRAIL receptor system and CD95 (69, 70). Specifically, an increase of apoptotic keratinocytes has been observed in CLE with concomitant increased epidermal expression of TRAIL-R1, CD95, and FADD (64, 69). Apoptotic keratinocytes contribute to the pathogenesis of CLE *via* the release of cellular debris, which results in a positive feedback loop of inflammation. Recent work by Kingsmore et al. (71) noted increased apoptotic mitochondrial gene signatures in DLE and lupus nephritis, suggesting a role for the intrinsic apoptotic pathway, and positive correlation with inflammatory cell signatures supports the intrinsic link of apoptosis with inflammation. Gene set expression analysis with microarray and RNA-sequencing of CLE skin and blood identified other genes, such as *GZMB*, *BAX*, and various caspases (*CASP8*/*10*) among others (1, 64, 72–75). Differential expression analysis of CLE lesional skin and blood skewed toward lesional skin, though apoptosis signatures were noted in both environments. Apoptosis and necroptosis pathways *via RIP3* are activated in interface dermatitis characteristic in CLE (76).

Downregulation of anti-apoptotic genes has also been identified in CLE. RANKL, a regulator of apoptosis, is notably absent from CLE skin (77). While TRAIL-R1 has been shown to be pro-apoptotic, TRAIL-R4 serves as a decoy receptor, blocking TRAIL-induced apoptosis, and has been shown to be downregulated in CLE relative to psoriasis and lichen planus (69, 78).

#### Complements, Lysosomes, and Proteosome Contribute to Impaired Clearance of Cell Debris in Cutaneous Lupus Erythematosus

The complement cascade plays a pivotal role in the opsonization of cells undergoing apoptosis to facilitate phagocytosis, and the timely clearance of cellular debris is important to prevent the generation of autoantibodies. Similarly, lysosomal and proteasomal clearance of cellular debris *via* proteolysis plays an important role and has been shown to be dysregulated in CLE. Skin gene expression of complement has been shown to be more dysregulated relative to blood gene expression in CLE (74, 79, 80). This is contrasted with the upregulation of cathepsins associated with lysosomes and proteasome-related genes in CLE peripheral blood relative to lesional skin (35, 74). The complex dysregulation of the systems involved in the clearance of cellular debris at a localized and systemic level highlights the complexity of the pathogenesis of CLE.

#### Fibrosis Is Likely Driven by TGF-β in Cutaneous Lupus Erythematosus

Of the CLE subtypes, DLE has been most associated with scarring lesions with associated fibrosis. Comparison of patients with DLE and SCLE using Ingenuity Pathway Analysis revealed pathways associated with fibrotic processes, and longitudinal microarray analysis of patients with DLE and SCLE revealed sustained elevations of *TGF-B1, TGF-BR1, SMAD3, MMP1, MMP9*, and *SERPINE1* (42). Interestingly, while TGF-β, a M2 macrophage-related protein, was noted to be overexpressed in DLE skin relative to normal skin in an independent experiment, other M2 macrophage-related genes, such as *CD206*, *CD209*, *FOLR2*, *IL10*, and arginase-1, were not differentially expressed (65). Taken together, *TGF-*β likely plays a key role in fibrogenesis in scarring DLE lesions, though the exact downstream mechanisms have yet to be fully defined.

## Conclusion

Our understanding of the underlying genetics governing the pathophysiology of CLE has greatly increased, thanks to advances in gene expression technology. Insights into the underlying genetic polymorphisms that predispose patients to CLE and knowledge of key dysregulated pathways in CLE afford the opportunity to develop targeted therapies for patients with CLE. Most recently, pathogenesis-directed therapy has focused on blockade of IFN receptors, such as anifrolumab (81). Other approaches, such as targeting the JAK/STAT pathway, are under investigation (82–84). The limitations of reviewed studies include small sample size, specific, non-generalizable cohorts, and technical limitations of gene expression profiling approaches, such as low resolution in microarray studies. Further studies using newer technologies, such as single-cell RNA sequencing, are warranted to define the genetic pathophysiology of CLE at greater resolution. Greater understanding of the underlying genetics of CLE can lead to further development of targeted therapies for CLE.

## Author Contributions

HC and BC conceived and designed the study. HC and GB acquired, analyzed, interpreted the data, and drafted the original manuscript. All authors contributed to critical revision of the manuscript for important intellectual content, read, and approved the submitted version.

## Conflict of Interest

BC was an investigator for Daavlin Corporation, Biogen Incorporated, and Pfizer Incorporated and consultant for Bristol Meyers Squibb, EMD Serono, Horizon Therapeutics, and Biogen Incorporated. The remaining authors declare that the research was conducted in the absence of any commercial or financial relationships that could be construed as a potential conflict of interest.

## Publisher’s Note

All claims expressed in this article are solely those of the authors and do not necessarily represent those of their affiliated organizations, or those of the publisher, the editors and the reviewers. Any product that may be evaluated in this article, or claim that may be made by its manufacturer, is not guaranteed or endorsed by the publisher.

## Supplementary Material

Supplementary Figure 1 Flow diagram of workflow for the literature review.

## Abbreviations

BAFF: B-cell activating factor
CCLE: chronic cutaneous lupus erythematosus
circRNAs: circular RNAs
CLE: cutaneous lupus erythematosus
DLE: discoid lupus erythematosus
HLA: human leukocyte antigen
IFN: interferon
IL: interleukin
iNOS: inducible nitric oxide synthase
LET: lupus erythematosus tumidus
lncRNAs: long non-coding RNAs
NLE, neonatal lupus erythematosus SLE: systemic lupus erythematosus
SNP: single nucleotide polymorphism
SYK: spleen tyrosine kinase.

## References

[1] Zhu JL Tran LT Smith M Zheng F Cai L James JA Modular Gene analysis reveals distinct molecular signatures for subsets of patients with cutaneous lupus erythematosus. *Brit J Dermatol.* (2021) 185:563–72. 10.1111/bjd.19800 33400293 PMC8255330

[2] Zeidi M Kim HJ Werth VP. Increased myeloid dendritic cells and tnf-alpha expression predicts poor response to hydroxychloroquine in cutaneous lupus erythematosus. *J Investig Dermatol.* (2019) 139:324–32. 10.1016/j.jid.2018.07.041 30227141 PMC7428867

[3] Fowler JF Callen JP Stelzer GT Cotter PK. Human histocompatibility antigen associations in patients with chronic cutaneous lupus erythematosus. *J Am Acad Dermatol.* (1985) 12:73–7. 3856581 10.1016/s0190-9622(85)70012-6

[4] Bielsa I Herrero C Ercilla G Collado A Font J Ingelmo M Immunogenetic findings in cutaneous lupus erythematosus. *J Am Acad Dermatol.* (1991) 25:251–7. 1918462 10.1016/0190-9622(91)70191-4

[5] Günther C Meurer M Stein A Viehweg A Lee-Kirsch M-A. Familial chilblain lupus–a monogenic form of cutaneous lupus erythematosus due to a heterozygous mutation in trex1. *Dermatology.* (2009) 219:162–6. 19478477 10.1159/000222430

[6] Millard T Kondeatis E Cox A Wilson A Grabczynska S Carey B A candidate gene analysis of three related photosensitivity disorders: cutaneous lupus erythematosus, polymorphic light eruption and actinic prurigo. *Br J Dermatol.* (2001) 145:229–36. 11531784 10.1046/j.1365-2133.2001.04339.x

[7] Partanen J Koskimies S Johansson E. C4 null phenotypes among lupus erythematosus patients are predominantly the result of deletions covering C4 and closely linked 21-hydroxylase a genes. *J Med Genet.* (1988) 25:387–91. 3260957 10.1136/jmg.25.6.387 PMC1050506

[8] Fischer GF Pickl WF Faé I Anegg B Milota S Volc-Platzer B. Association between chronic cutaneous lupus erythematosus and Hla class II alleles. *Hum Immunol.* (1994) 41:280–4. 7883595 10.1016/0198-8859(94)90046-9

[9] Miyagawa S Shinohara K Fujita T Kidoguchi K-I Fukumoto T Hashimoto K Neonatal lupus erythematosus: analysis of Hla class II Alleles in mothers and siblings from seven Japanese families. *J Am Acad Dermatol.* (1997) 36:186–90. 9039166 10.1016/s0190-9622(97)70278-0

[10] Lopez-Tello A Rodríguez-Carreón A Jurado F Yamamoto-Furusho J Castillo-Vázquez M Chávez-Muñoz C Association of Hla-Drb1* 16 with chronic discoid lupus erythematosus in Mexican mestizo patients. *Clin Exp Dermatol Exp Dermatol.* (2007) 32:435–8. 10.1111/j.1365-2230.2007.02391.x 17376212

[11] Racila DM Sontheimer CJ Sheffield A Wisnieski JJ Racila E Sontheimer RD. Homozygous single nucleotide polymorphism of the complement C1qa gene is associated with decreased levels of C1q in patients with subacute cutaneous lupus erythematosus. *Lupus.* (2003) 12:124–32. 10.1191/0961203303lu329oa 12630757

[12] Jlajla H Sellami MK Sfar I Laadhar L Zerzeri Y Abdelmoula MS New C1q mutation in a tunisian family. *Immunobiology.* (2014) 219:241–6. 10.1016/j.imbio.2013.10.010 24331529

[13] Callen JP Hodge SJ Kulick KB Stelzer G Buchino JJ. Subacute cutaneous lupus erythematosus in multiple members of a family with C2 deficiency. *Arch Dermatol.* (1987) 123:66–70. 3467658

[14] Belin DC Bordwell BJ Einarson ME McLean RH Weinstein A Yunis EJ Familial discoid lupus erythematosus associated with heterozygote C2 deficiency. *Arthritis Rheum.* (1980) 23:898–903. 10.1002/art.1780230804 6902670

[15] Levy SB Pinnell SR Meadows L Snyderman R Ward FE. Hereditary C2 deficiency associated with cutaneous lupus erythematosus: clinical, laboratory, and genetic studies. *Arch Dermatol.* (1979) 115:57–61. 760659

[16] Agnello V Gell J Tye MJ. Partial genetic deficiency of the C4 component of complement in discoid lupus erythematosus and Urticaria/Angioedema. *J Am Acad Dermatol.* (1983) 9:894–8. 10.1016/s0190-9622(83)70205-7 6643787

[17] Günther C Hillebrand M Brunk J Lee-Kirsch MA. Systemic involvement in trex1-associated familial chilblain lupus. *J Am Acad Dermatol.* (2013) 69:e179–81. 24034389 10.1016/j.jaad.2013.04.020

[18] Günther C Berndt N Wolf C Lee-Kirsch MA. Familial chilblain lupus due to a novel mutation in the exonuclease III domain of 3’ repair exonuclease 1 (Trex1). *JAMA Dermatol.* (2015) 151:426–31. 25517357 10.1001/jamadermatol.2014.3438

[19] Rice G Newman WG Dean J Patrick T Parmar R Flintoff K Heterozygous mutations in Trex1 cause familial chilblain lupus and dominant aicardi-goutieres syndrome. *Am J Hum Genet.* (2007) 80:811–5. 17357087 10.1086/513443 PMC1852703

[20] Ravenscroft JC Suri M Rice GI Szynkiewicz M Crow YJ. Autosomal dominant inheritance of a heterozygous mutation in samhd1 causing familial chilblain lupus. *Am J Med Genet Part A.* (2011) 155:235–7. 10.1002/ajmg.a.33778 21204240

[21] Linggonegoro D Song H Jones K Lee P Schmidt B Vleugels R Familial chilblains lupus in a child with heterozygous mutation in samhd1 and normal interferon signature. *Br J Dermatol.* (2021) 185:650–2. 33887057 10.1111/bjd.20400

[22] Popovic K Ek M Espinosa A Padyukov L Harris HE Wahren-Herlenius M Increased expression of the novel proinflammatory cytokine high mobility group box chromosomal protein 1 in skin lesions of patients with lupus erythematosus. *Arthritis Rheumat.* (2005) 52:3639–45. 16255056 10.1002/art.21398

[23] Suárez A López P Mozo L Gutiérrez C. Differential effect of IL10 and Tnfα genotypes on determining susceptibility to discoid and systemic lupus erythematosus. *Ann Rheumat Dis.* (2005) 64:1605–10. 15800006 10.1136/ard.2004.035048 PMC1755257

[24] Skonieczna K Czajkowski R Kaszewski S Gawrych M Jakubowska A Grzybowski T. Genetic similarities and differences between discoid and systemic lupus erythematosus patients within the polish population. *Adv Dermatol Allergol Post?py Dermatol Alergol.* (2017) 34:228. 10.5114/pdia.2017.67479 PMC5471372 28670251

[25] Järvinen TM Hellquist A Koskenmies S Einarsdottir E Panelius J Hasan T Polymorphisms of the itgam gene confer higher risk of discoid cutaneous than of systemic lupus erythematosus. *PLoS One.* (2010) 5:e14212. 10.1371/journal.pone.0014212 21151989 PMC2996302

[26] Kunz M Konig IR Schillert A Kruppa J Ziegler A Grallert H Genome-wide association study identifies new susceptibility loci for cutaneous lupus erythematosus. *Exp Dermatol.* (2015) 24:510–5. 10.1111/exd.12708 25827949

[27] Skonieczna K Woźniacka A Czajkowski R Styczyñski J Krenska A Robak E X-linked Tlr7 gene polymorphisms are associated with diverse immunological conditions but not with discoid lupus erythematosus in Polish patients. *Adv Dermatol Allergol.* (2018) 35:26. 10.5114/pdia.2017.69984 PMC5872239 29599669

[28] Nemer G El-Hachem N Eid E Hamie L Bardawil T Khalil S A novel Traf3ip2 variant causing familial scarring alopecia with mixed features of discoid lupus erythematosus and folliculitis decalvans. *Clin Genet.* (2020) 98:116–25. 32350852 10.1111/cge.13767

[29] Järvinen TM Hellquist A Koskenmies S Einarsdottir E Koskinen LL Jeskanen L Tyrosine kinase 2 and interferon regulatory factor 5 polymorphisms are associated with discoid and subacute cutaneous lupus erythematosus. *Exp Dermatol.* (2010) 19:123–31. 19758313 10.1111/j.1600-0625.2009.00982.x

[30] König N Fiehn C Wolf C Schuster M Costa EC Tüngler V Familial chilblain lupus due to a gain-of-function mutation in sting. *Ann Rheumat Dis.* (2017) 76:468–72. 27566796 10.1136/annrheumdis-2016-209841

[31] Weller R Schwentker A Billiar TR Vodovotz Y. Autologous nitric oxide protects mouse and human keratinocytes from ultraviolet B radiation-induced apoptosis. *Am J Physiol Cell Physiol.* (2003) 284:C1140–8. 10.1152/ajpcell.00462.2002 12676653

[32] Suschek CV Krischel V Bruch-Gerharz D Berendji D Krutmann J Kroncke KD Nitric oxide fully protects against Uva-induced apoptosis in tight correlation with Bcl-2 up-regulation. *J Biol Chem.* (1999) 274:6130–7. 10.1074/jbc.274.10.6130 10037696

[33] Kuhn A Fehsel K Lehmann P Krutmann J Ruzicka T Kolb-Bachofen V. Aberrant timing in epidermal expression of inducible nitric oxide synthase after Uv irradiation in cutaneous lupus erythematosus. *J Invest Dermatol.* (1998) 1:149–53. 10.1046/j.1523-1747.1998.00253.x 9665402

[34] Meller S Winterberg F Gilliet M Muller A Lauceviciute I Rieker J Ultraviolet radiation-induced injury, chemokines, and leukocyte recruitment - an amplification cycle triggering cutaneous lupus erythematosus. *Arthritis Rheum US.* (2005) 52:1504–16. 10.1002/art.21034 15880822

[35] Katayama S Panelius J Koskenmies S Skoog T Mahonen K Kisand K Delineating the healthy human skin Uv response and early induction of interferon pathway in cutaneous lupus erythematosus. *J Invest Dermatol.* (2019) 139:2058–61e4. 10.1016/j.jid.2019.02.035 30974166

[36] Xiang MM Chen Q Feng Y Wang YL Wang J Liang J Bioinformatic analysis of key biomarkers and immune filtration of skin biopsy in discoid lupus erythematosus. *Lupus.* (2021) 30:807–17. 10.1177/0961203321992434 33530816

[37] Toro JR Finlay D Dou X Zheng SC LeBoit PE Connolly MK. Detection of type 1 cytokines in discoid lupus erythematosus. *Arch Dermatol.* (2000) 136:1497–501. 10.1001/archderm.136.12.1497 11115160

[38] Stein LF Saed GM Fivenson DP. T-cell cytokine network in cutaneous lupus erythematosus. *J Am Acad Dermatol.* (1997) 36:191–6. 10.1016/s0190-9622(97)70279-2 9039167

[39] Denfeld RW Kind P Sontheimer RD Schopf E Simon JC. In Situ expression of B7 and Cd28 receptor families in skin lesions of patients with lupus erythematosus. *Arthritis Rheum US.* (1997) 40:814–21. 10.1002/art.1780400507 9153541

[40] Jabbari A Suarez-Farinas M Fuentes-Duculan J Gonzalez J Cueto I Franks AG Jr. Dominant Th1 and minimal Th17 skewing in discoid lupus revealed by transcriptomic comparison with psoriasis. *J Invest Dermatol.* (2014) 134:87–95. 10.1038/jid.2013.269 23771123 PMC3858414

[41] Nickles MA Huang K Chang YS Tsoukas MM Sweiss NJ Perkins DL Gene co-expression networks identifies common hub genes between cutaneous sarcoidosis and discoid lupus erythematosus. *Front Med.* (2020) 7:606461. 10.3389/fmed.2020.606461 33324666 PMC7724034

[42] Sole C Gimenez-Barcons M Ferrer B Ordi-Ros J Cortes-Hernandez J. Microarray study reveals a transforming growth factor-beta-dependent mechanism of fibrosis in discoid lupus erythematosus. *Br J Dermatol.* (2016) 175:302–13. 10.1111/bjd.14539 26972571

[43] Tsoi LC Gharaee-Kermani M Berthier CC Nault T Hile GA Estadt SN Il18-containing 5-gene signature distinguishes histologically identical dermatomyositis and lupus erythematosus skin lesions. *JCI Insight.* (2020) 5:20. 10.1172/jci.insight.139558 32644977 PMC7455118

[44] Dong Q Chen K Xie J Han H Feng Y Lu J Identification of key genes and pathways in discoid lupus skin via bioinformatics analysis. *Medicine.* (2021) 100:e25433. 10.1097/MD.0000000000025433 33879674 PMC8078291

[45] Scholtissek B Zahn S Maier J Klaeschen S Braegelmann C Hoelzel M Immunostimulatory endogenous nucleic acids drive the lesional inflammation in cutaneous lupus erythematosus. *J Invest Dermatol.* (2017) 137:1484–92. 10.1016/j.jid.2017.03.018 28351661

[46] Merola JF Wang W Wager CG Hamann S Zhang X Thai A Rna tape sampling in cutaneous lupus erythematosus discriminates affected from unaffected and healthy volunteer skin. *Lupus Sci Med.* (2021) 8:428. 10.1136/lupus-2020-000428 33658303 PMC7931768

[47] Fetter T Smith P Guel T Braegelmann C Bieber T Wenzel J. Selective janus kinase 1 inhibition is a promising therapeutic approach for lupus erythematosus skin lesions. *Front.* (2020) 11:344. 10.3389/fimmu.2020.00344 32194562 PMC7064060

[48] Calugareanu A Grolleau C Le Buanec H Chasset F Jachiet M Battistella M clinical efficacy of selective Jak1 inhibition and transcriptome analysis of chronic discoid lupus erythematosus. *J Eur Acad Dermatol Venereol.* (2022) 36:e308–10. 10.1111/jdv.17839 34839559

[49] Taylor KR Gallo RL. Glycosaminoglycans and their proteoglycans: host-associated molecular patterns for initiation and modulation of inflammation. *FASEB J.* (2006) 20:9–22. 10.1096/fj.05-4682rev 16394262

[50] Chang LM Maheshwari P Werth S Schaffer L Head SR Kovarik C Identification and molecular analysis of glycosaminoglycans in cutaneous lupus erythematosus and dermatomyositis. *J Histochem Cytochem.* (2011) 59:336–45. 10.1369/0022155410398000 21378287 PMC3201158

[51] Yang J Li Q Yang X Li M. Interleukin-9 is associated with elevated anti-double-stranded DNA antibodies in lupus-prone mice. *Mol Med.* (2015) 21:364–70. 10.2119/molmed.2014.00237 25902303 PMC4534470

[52] Xie Y Liu B Wu Z. Increased interleukin-9 levels in skin lesions from cutaneous lupus erythematosus patients may predict the progression to systemic lupus erythematosus. *J Dermatol Sci.* (2021) 101:78–80. 10.1016/j.jdermsci.2020.10.016 33172731

[53] Chong BF Tseng LC Kim A Miller RT Yancey KB Hosler GA. Differential expression of baff and its receptors in discoid lupus erythematosus patients. *J Dermatol Sci.* (2014) 73:216–24. 10.1016/j.jdermsci.2013.11.007 24315762 PMC3946198

[54] Mocsai A Ruland J Tybulewicz VL. The syk tyrosine kinase: a crucial player in diverse biological functions. *Nat Rev Immunol.* (2010) 10:387–402. 10.1038/nri2765 20467426 PMC4782221

[55] Braegelmann C Holzel M Ludbrook V Dickson M Turan N Ferring-Schmitt S Spleen tyrosine kinase (Syk) is a potential target for the treatment of cutaneous lupus erythematosus patients. *Exp Dermatol.* (2016) 25:375–9. 10.1111/exd.12986 26910509

[56] Vorwerk G Zahn S Bieber T Wenzel J. Nkg2d and its ligands as cytotoxic factors in cutaneous lupus erythematosus. *Exp Dermatol.* (2021) 30:847–52. 10.1111/exd.14311 33687107

[57] Guerra N Pestal K Juarez T Beck J Tkach K Wang L A selective role of Nkg2d in inflammatory and autoimmune diseases. *Clin Immunol.* (2013) 149:432–9. 10.1016/j.clim.2013.09.003 24211717 PMC3868205

[58] Tsoi LC Hile GA Berthier CC Sarkar MK Reed TJ Liu J Hypersensitive Ifn responses in lupus keratinocytes reveal key mechanistic determinants in cutaneous lupus. *J Immunol.* (2019) 202:2121–30. 10.4049/jimmunol.1800650 30745462 PMC6424612

[59] Xuan J Xiong YY Shi LJ Aramini B Wang HY. Do lncrnas and circrnas expression profiles influence discoid lupus erythematosus progression?-a comprehensive analysis. *Ann Translat Med.* (2019) 7:10. 10.21037/atm.2019.12.10 32042744 PMC6990042

[60] Blomberg S Eloranta ML Cederblad B Nordlin K Alm GV Ronnblom L. Presence of cutaneous interferon-alpha producing cells in patients with systemic lupus erythematosus. *Lupus.* (2001) 10:484–90. 10.1191/096120301678416042 11480846

[61] Wongpiyabovorn J Ruchusatsawat K Onganantapong Y Sintupak W Hirankarn N. Interferon alpha Mrna level and subtypes in lesion and non-lesion from discoid lupus erythematosus patients without systemic lupus erythematosus. *Asian Biomed.* (2011) 5:643–7. 10.5372/1905-7415.0505.085

[62] Sirobhushanam S Parsa N Reed TJ Berthier CC Sarkar MK Hile GA Staphylococcus aureus colonization is increased on lupus skin lesions and is promoted by Ifn-mediated barrier disruption. *J Investig Dermatol.* (2020) 140:1066.e–74.e. 10.1016/j.jid.2019.11.016 31877319 PMC7183889

[63] Stannard JN Reed TJ Myers E Lowe L Sarkar MK Xing XY Lupus skin is primed for IL-6 inflammatory responses through a keratinocyte-mediated autocrine type I interferon loop. *J Investig Dermatol.* (2017) 137:115–22. 10.1016/j.jid.2016.09.008 27646883 PMC5183476

[64] Sarkar MK Hile GA Tsoi LC Xing X Liu J Liang Y Photosensitivity and type I Ifn responses in cutaneous lupus are driven by epidermal-derived interferon Kappa. *Ann Rheum Dis.* (2018) 77:1653–64. 10.1136/annrheumdis-2018-213197 30021804 PMC6185784

[65] Chong BF Tseng LC Hosler GA Teske NM Zhang S Karp DR A subset of Cd163+ macrophages displays mixed polarizations in discoid lupus skin. *Arthritis Res Ther.* (2015) 17:324. 10.1186/s13075-015-0839-3 26568320 PMC4644297

[66] Flier J Boorsma DM van Beek PJ Nieboer C Stoof TJ Willemze R Differential expression of Cxcr3 targeting chemokines Cxcl10, Cxcl9, and Cxcl11 in different types of skin inflammation. *J Pathol.* (2001) 194:398–405. 10.1002/1096-9896(200108)194:43.0.co;2-s 11523046

[67] Gambichler T Genc Z Skrygan M Scola N Tigges C Terras S Cytokine and chemokine ligand expression in cutaneous lupus erythematosus. *Eur J Dermatol.* (2012) 22:319–23. 10.1684/ejd.2012.1725 22562806

[68] Gao ZY Su LC Wu QC Sheng JE Wang YL Dai YF Bioinformatics analyses of gene expression profile identify key genes and functional pathways involved in cutaneous lupus erythematosus. *Clin Rheumatol.* (2022) 41:437–52. 10.1007/s10067-021-05913-2 34553293

[69] Toberer F Sykora J Gottel D Hartschuh W Werchau S Enk A Apoptotic signal molecules in skin biopsies of cutaneous lupus erythematosus: analysis using tissue microarray. *Exp Dermatol.* (2013) 22:656–9. 10.1111/exd.12216 24079735

[70] Zahn S Rehkamper C Ferring-Schmitt S Bieber T Tuting T Wenzel J. Interferon-alpha stimulates trail expression in human keratinocytes and peripheral blood mononuclear cells: implications for the pathogenesis of cutaneous lupus erythematosus. *Br J Dermatol.* (2011) 165:1118–23. 10.1111/j.1365-2133.2011.10479.x 21711324

[71] Kingsmore KM Bachali P Catalina MD Daamen AR Heuer SE Robl RD Altered expression of genes controlling metabolism characterizes the tissue response to immune injury in lupus. *Sci.* (2021) 11:14789. 10.1038/s41598-021-93034-w 34285256 PMC8292402

[72] Ko WC Li L Young TR McLean-Mandell RE Deng AC Vanguri VK Gene expression profiling in the skin reveals strong similarities between subacute and chronic cutaneous lupus that are distinct from lupus nephritis. *J Invest Dermatol.* (2021) 141:2808–19. 10.1016/j.jid.2021.04.030 34153327

[73] Dey-Rao R Sinha AA. Genome-wide transcriptional profiling of chronic cutaneous lupus erythematosus (Ccle) peripheral blood identifies systemic alterations relevant to the skin manifestation. *Genomics.* (2015) 105:90–100. 10.1016/j.ygeno.2014.11.004 25451738

[74] Dey-Rao R Sinha AA. In silico analyses of skin and peripheral blood transcriptional data in cutaneous lupus reveals Ccr2-a novel potential therapeutic target. *Front Immunol.* (2019) 10:640. 10.3389/fimmu.2019.00640 30984198 PMC6450170

[75] Dey-Rao R Smith JR Chow S Sinha AA. Differential gene expression analysis in Ccle lesions provides new insights regarding the genetics basis of skin Vs. systemic disease. *Genomics.* (2014) 104:144–55. 10.1016/j.ygeno.2014.06.003 24956118

[76] Lauffer F Jargosch M Krause L Garzorz-Stark N Franz R Roenneberg S Type I immune response induces keratinocyte necroptosis and is associated with interface dermatitis. *J Invest Dermatol.* (2018) 138:1785–94. 10.1016/j.jid.2018.02.034 29526761

[77] Toberer F Sykora J Gottel D Ruland V Hartschuh W Enk A Tissue microarray analysis of rankl in cutaneous lupus erythematosus and psoriasis. *Exp Dermatol.* (2011) 20:600–2. 10.1111/j.1600-0625.2011.01303.x 21692859

[78] Nguyen V Cudrici C Zernetkina V Niculescu F Rus H Drachenberg C Trail, Dr4 and Dr5 are upregulated in kidneys from patients with lupus nephritis and exert proliferative and proinflammatory effects. *Clin Immunol.* (2009) 132:32–42. 10.1016/j.clim.2009.02.011 19349211 PMC3734543

[79] Alase A Shalbaf M Berekmeri A Yusof MYM Botchkareva N Goodfield M Analysis of noninvasively collected hair follicles may be sufficient for the diagnosis of chronic discoid lupus erythematosus of the scalp. *Br J Dermatol.* (2019) 180:E205–6.

[80] Shalbaf M Alase AA Berekmeri A Md Yusof MY Pistolic J Goodfield MJ Plucked hair follicles from patients with chronic discoid lupus erythematosus show a disease-specific molecular signature. *Lupus Sci Med.* (2019) 6:e000328. 10.1136/lupus-2019-000328 31413850 PMC6667780

[81] Morand EF Furie R Tanaka Y Bruce IN Askanase AD Richez C Trial of anifrolumab in active systemic lupus erythematosus. *N Engl J Med.* (2020) 382:211–21. 10.1056/NEJMoa1912196 31851795

[82] Wallace DJ Furie RA Tanaka Y Kalunian KC Mosca M Petri MA Baricitinib for systemic lupus erythematosus: a double-blind, randomised, placebo-controlled, phase 2 trial. *Lancet.* (2018) 392:222–31. 10.1016/S0140-6736(18)31363-1 30043749

[83] Werth VP Fleischmann R Robern M Touma Z Tiamiyu I Gurtovaya O Filgotinib or lanraplenib in moderate to severe cutaneous lupus erythematosus: a phase 2, randomised, double-blind, placebo-controlled study. *Rheumatology.* (2021) 2021:keab685. 10.1093/rheumatology/keab685 34498056 PMC9157055

[84] Klaeschen AS Wolf D Brossart P Bieber T Wenzel J. Jak inhibitor ruxolitinib inhibits the expression of cytokines characteristic of cutaneous lupus erythematosus. *Exp Dermatol.* (2017) 26:728–30. 10.1111/exd.13253 27892610

