# Supplementary material for: The Genetic Landscape of Cutaneous Lupus Erythematosus
Source: Front Med (Lausanne). 2022 Jun 2;9:916011. doi: 10.3389/fmed.2022.916011 (PMC9201079; doi:10.3389/fmed.2022.916011)
Supplement: Supplementary file 1 [file Table_1.docx]

**Supplementary Table 1. Search strategies for MEDLINE and Embase database via Ovid.** Number of identified articles are portrayed on each line in parentheses for each search term.

| MEDLINE | Embase |
| --- | --- |
| 1 exp Lupus Erythematosus, Cutaneous/ (5365)  2 cutaneous lupus erythematosus.mp. (1624)  3 exp Panniculitis, Lupus Erythematosus/ (290)  4 lupus erythematosus panniculitis.mp. (81)  5 exp Lupus Erythematosus, Discoid/ (3300)  6 discoid lupus.mp. (1638)  7 acute cutaneous lupus erythematosus.mp. (44)  8 chronic cutaneous lupus erythematosus.mp. (145)  9 chronic lupus.mp. (254)  10 intermittent cutaneous lupus erythematosus.mp. (2)  11 lupus erythematosus tumidus.mp. (75)  12 tumid lupus.mp. (32)  13 subacute cutaneous lupus erythematosus.mp. (606)  14 subacute lupus.mp. (121)  15 chilblain lupus.mp. (153)  16 exp Disease Susceptibility/ or exp Genetic Markers/ or exp Genome-Wide Association Study/ or exp Genetic Heterogeneity/ or exp Genetic Linkage/ (305499)  17 exp Sequence Analysis, RNA/ (27181)  18 exp Microarray Analysis/ or exp Gene Expression Profiling/ (207295)  19 gene expression regulation.mp. or exp Gene Expression Regulation/ (1092286)  20 exp RNA, Messenger/ (431130)  21 exp Multigene Family/ (47657)  22 exp Genetic Association Studies/ (65604)  23 exp Genetic Predisposition to Disease/ (151063)  24 exp Polymorphism, Genetic/ or exp Polymorphism, Single Nucleotide/ (291861)  25 exp Histocompatibility Antigens/ge [Genetics] (57504)  26 gene association study.mp. (236)  27 exp Gene Expression Profiling/ (150667)  28 exp Gene Expression Regulation/ (1091515)  29 exp Panniculitis, Lupus Erythematosus/ge [Genetics] (10)  30 exp Lupus Erythematosus, Cutaneous/ge [Genetics] (249)  31 exp Genetic Testing/ (50970)  32 exp Whole Exome Sequencing/ (6321)  33 exp Transcriptome/ (54699)  34 exp Gene Regulatory Networks/ (28170)  35 exp Transcription Factors/ (641608)  36 exp Mutation/ge [Genetics] (112415)  37 exp RNA/ge [Genetics] (433628)  38 exp DNA/ge [Genetics] (309770)  39 exp Lupus Erythematosus, Discoid/ge [Genetics] (117)  40 1 or 2 or 3 or 4 or 5 or 6 or 7 or 8 or 9 or 10 or 11 or 12 or 13 or 14 or 15 (6222)  41 16 or 17 or 18 or 19 or 20 or 21 or 22 or 23 or 24 or 25 or 26 or 27 or 28 or 29 or 30 or 31 or 32 or 33 or 34 or 35 or 36 or 37 or 38 or 39 (2654699)  42 40 and 41 (421)  43 limit 42 to (english language and humans) (361)  44 limit 43 to "review articles" (68)  45 43 not 44 (293) | 1 cutaneous lupus erythematosus.mp. or exp skin lupus erythematosus/ (7099)  2 lupus erythematosus panniculitus.mp. (0)  3 discoid lupus erythematosus.mp. or exp discoid lupus erythematosus/ (3918)  4 acute cutaneous lupus erythematosus.mp. (91)  5 chronic cutaneous lupus erythematosus.mp. (260)  6 chronic lupus.mp. (160)  7 intermittent cutaneous lupus erythematosus.mp. (5)  8 lupus erythematosus tumidus.mp. (137)  9 tumid lupus.mp. (85)  10 subacute cutaneous lupus erythematosus.mp. (879)  11 subacute lupus.mp. (149)  12 chilblain lupus.mp. (260)  13 exp disease predisposition/et [Etiology] (281)  14 exp genetic marker/ (114144)  15 exp genome-wide association study/ (35301)  16 exp genetic heterogeneity/ (526153)  17 exp genetic linkage/ (81323)  18 exp RNA sequencing/ (44134)  19 exp multigene family/ (33374)  20 exp genetic association study/ (39082)  21 exp genetic predisposition/ (185279)  22 exp genetic polymorphism/ (487525)  23 exp single nucleotide polymorphism/ (213238)  24 exp histocompatibility antigen/ (168142)  25 gene association study.mp. (392)  26 exp genetic association/ (227283)  27 exp DNA polymorphism/ (323617)  28 exp gene expression profiling/ (127275)  29 exp gene expression/ (1832134)  30 exp gene expression regulation/ (294729)  31 exp skin lupus erythematosus/cn, et [Congenital Disorder, Etiology] (517)  32 exp discoid lupus erythematosus/cn, et [Congenital Disorder, Etiology] (168)  33 exp genetic screening/ (103201)  34 exp whole exome sequencing/ (33299)  35 exp genome analysis/ (41905)  36 exp transcriptome/ (82787)  37 exp transcriptome sequencing/ (3231)  38 exp gene regulatory network/ (20790)  39 exp transcription factor/ (921991)  40 exp mutation/ (1281569)  41 exp RNA/ (1360024)  42 exp DNA/ (983003)  43 1 or 2 or 3 or 4 or 5 or 6 or 7 or 8 or 9 or 10 or 11 or 12 (7746)  44 13 or 14 or 15 or 16 or 17 or 18 or 19 or 20 or 21 or 22 or 23 or 24 or 25 or 26 or 27 or 28 or 29 or 30 or 31 or 32 or 33 or 34 or 35 or 36 or 37 or 38 or 39 or 40 or 41 or 42 (5102187)  45 43 and 44 (1409)  46 45 not ((exp animal/ or nonhuman/) not exp human/) (1346)  47 limit 46 to english language (1252)  48 limit 46 to (english language and "review") (292)  49 47 not 48 (960) |
