# Supplementary figures and images for: The Genetic Landscape of Cutaneous Lupus Erythematosus
Source: Front Med (Lausanne). 2022 Jun 2;9:916011. doi: 10.3389/fmed.2022.916011 (PMC9201079; doi:10.3389/fmed.2022.916011)

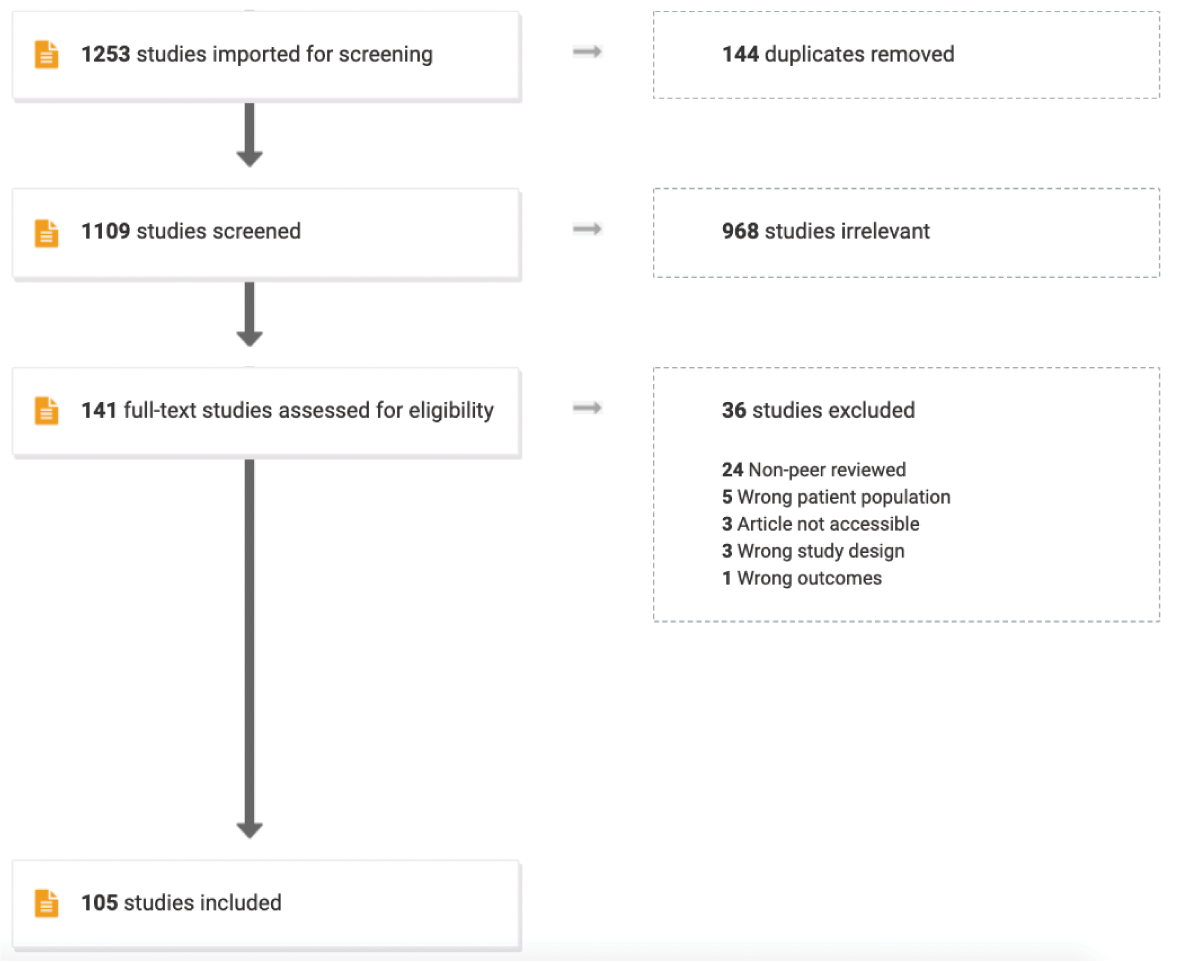

Supplement: Supplementary Figure 1 — Flow diagram of workflow for the literature review. [file Image_1.TIF]
